# Long non-coding RNA-SNHG7 acts as a target of miR-34a to increase GALNT7 level and regulate PI3K/Akt/mTOR pathway in colorectal cancer progression

**DOI:** 10.1186/s13045-018-0632-2

**Published:** 2018-07-03

**Authors:** Yang Li, Changqian Zeng, Jialei Hu, Yue Pan, Yujia Shan, Bing Liu, Li Jia

**Affiliations:** 1 College of Laboratory Medicine, Dalian Medical University, Dalian, 116044 Liaoning Province China; 2 Medical College, Dalian University, Dalian, 116622 Liaoning Province China

**Keywords:** lncRNA-SNHG7, miR-34a, GALNT7, Progression

## Abstract

**Background:**

Colorectal cancer (CRC) arises in a multistep molecular network process, which is from either discrete genetic perturbation or epigenetic dysregulation. The long non-coding RNAs (lncRNAs), emerging as key molecules in human malignancy, has become one of the hot topics in RNA biology. Aberrant *O*-glycosylation is a well-described hallmark of many cancers. GALNT7 acts as a glycosyltransferase in protein *O*-glycosylation, involving in the occurrence and development of CRC.

**Methods:**

The microarrays were used to survey the lncRNA and mRNA expression profiles of primary CRC cell line SW480 and metastatic CRC cell line SW620. Cell proliferation, migration, invasion, and apoptosis were assayed. Xenograft mouse models were used to determine the role of lncRNA-SNHG7 in CRC in vivo. In addition, CNC analysis and competing endogenous analysis were used to detect differential SNHG7 and relational miRNAs expression in CRC cell lines.

**Results:**

SNHG7 expression showed a high fold (SW620/SW480) in CRC microarrays. The CRC patients with high expression of SNHG7 had a significantly poor prognosis. Furthermore, SNHG7 promoted CRC cell proliferation, metastasis, mediated cell cycle, and inhibited apoptosis. SNHG7 and GALNT7 were observed for co-expression by CNC analysis, and a negative correlation of SNHG7 and miR-34a were found by competing endogenous RNA (ceRNA) analysis. Further results indicated that SNHG7 facilitated the proliferation and metastasis as a competing endogenous RNA to regulate GALNT7 expression by sponging miR-34a in CRC cell lines. SNHG7 also played the oncogenic role in regulating PI3K/Akt/mTOR pathway by competing endogenous miR-34a and GALNT7.

**Conclusion:**

The CRC-related SNHG7 and miR-34a might be implicated in CRC progression via GALNT7, suggesting the potential usage of SNHG7/miR-34a/GALNT7 axis in CRC treatment.

**Electronic supplementary material:**

The online version of this article (10.1186/s13045-018-0632-2) contains supplementary material, which is available to authorized users.

## Background

Colorectal cancer (CRC), a high-risk digestive tract tumor, is one of the most frequent malignant tumors worldwide [1]. The pathogenesis of CRC involves multiple factors, including environmental and genetic variables, while detailed molecular mechanisms remain unclear [2]. Hence, a better understanding of the mechanisms and finding predictive biomarkers are urgently needed to detect CRC.

Long non-coding RNAs (lncRNAs) are over 200 nucleotides in length without protein-coding capacity. Mounting evidence demonstrates that lncRNAs may be emerged as essential regulators in many biological processes [3]. MicroRNAs (miRNAs, 20–25 nt) bind to the 3′-untranslated region (3′-UTR) of mRNA, catalyzed by the RNA-induced silencing complex (RISC), which subsequently cause degradation of the target mRNA or inhibition of its translation [4]. Recent literature has documented that lncRNAs enrich in the cytoplasm typically participate in post-transcriptional regulation by interacting with miRNAs or mRNAs [5], and play an active role in regulating miRNA availability within the cell and form regulatory networks [6].

SNHG7 (small nucleolar RNA host gene 7) is one of the recognized lncRNAs, which is located on chromosome 9q34.3, with a length of 2176 bp [7]. SNHG7 promotes the proliferation, migration, and invasion and inhibits apoptosis in many cancers, such as malignant pleural mesothelioma [8], breast cancer [9], chromophobe renal cell carcinoma [10], and lung cancer [11]. However, the clinical significance and biological mechanisms of SNHG7 in the progression of CRC remain largely to be elucidated.

Glycosylation is a common and highly diverse form of protein modification [12] and plays a pivotal role in malignancy. As a member of the acetylgalactosaminyltransferase family, GALNT7 materializes a certain biological effect by regulating the interaction between tumor cells and the extracellular environment. GALNT7 expression is a well-described hallmark of many cancers such as cervical cancer, pancreatic cancer, and laryngocarcinoma [13–15]. Recently, promising evidence has shown that the mRNA encoded by competing endogenous RNA (ceRNA) genes could be involved in distinct biological processes. Exploration of the function and involvement of lncRNA-miRNA-mRNA crosstalk may become a key development in exploring the molecular mechanisms of cancer.

In the present study, differences between the lncRNA and mRNA expression profiles of CRC cell lines were assessed in lncRNA microarray. High level of lncRNA-SNHG7 was correlated with tumor size, lymphatic metastasis, distant metastasis, and tumor stage. In addition, we investigated whether SNHG7 directly bound to miR-34a to de-repress the target gene of GALNT7 and participated in the regulation of CRC progression via PI3K/Akt/mTOR pathway. Our findings provided further evidence that SNHG7 regulated GALNT7 by sponging miR-34a and contributed to CRC progression, which might provide novel insights into the function of lncRNA-driven in CRC.

## Methods

### Clinical samples, cell lines, and culture condition

Two independent cohorts were enrolled. Cohort 1: Fresh CRC and adjacent tissues were collected from 53 patients between March 2015 and January 2018. Cohort 2: Liquid nitrogen storage tissues from 70 CRC patients who initially underwent tumor between January 2011 and May 2012. Tumor tissues and adjacent tissues (5 cm from the tumor edge) were obtained at the First Affiliated Hospital of Dalian Medical University (Dalian, China). None of the patients received any chemotherapy or radiation treatment prior to the surgery. The study and its informed consent have been examined and certified by the Ethics Committee.

CRC cell lines caco2, SW480, SW620, Hct116, and LoVo, and human normal colon epithelial cell line (FHC) were purchased from KeyGEN Company (Nanjing, China). The primary CRC cell line was established from a primary carcinoma of the CRC. Cells were cultured in 90% DMEM (Gibco) supplemented with 10% heat-inactivated fetal bovine serum (Gibco) and 1% penicillin–streptomycin (HyClone, Logan, UT, USA) at 37 °C with 5% CO_2_.

### RNA labeling and array hybridization

Labeled sample and array hybridization were analyzed according to the Agilent One-Color Microarray-Based Gene Expression Analysis protocol (Agilent Technology). The rRNA was removed, and mRNA was purified from total RNA (mRNA-ONLY™ Eukaryotic mRNA Isolation Kit, Epicentre). Then, each specimen was amplified and transcribed into fluorescent cRNA (Arraystar Flash RNA Labeling Kit, Arraystar). The labeled cRNAs were purified using RNeasy Mini Kit (Qiagen). The concentration and specific activity of the labeled cRNAs (pmol Cy3/μg cRNA) were determined by NanoDrop ND-1000. Each labeled cRNA (1 μg) was fragmented by adding 5 μl 10 × Blocking Agent and 1 μl of 25 × Fragmentation Buffer. The mixture was heated at 60 °C for 30 min, and 25 μl 2 × GE Hybridization buffer was added. Hybridization solution (50 μl) was applied and assembled to the lncRNA expression microarray slide. The slides were treated for 17 h at 65 °C in an Agilent Hybridization Oven. The hybridized arrays were scanned by the Agilent DNA Microarray Scanner (part number G2505C).

### Microarray and computational analysis

LncRNA expression profiles of SW480 and SW620 (*n* = 3/group) were used to synthesize double-stranded cDNA and hybridized to the 8x60K. RNA quantity and quality were measured by NanoDrop ND-1000. RNA integrity was assessed by standard denaturing agarose gel electrophoresis or Agilent 2100 Bioanalyzer. Arraystar Human LncRNA Microarray V4.0 was designed for the global profiling of human lncRNAs and protein-coding transcripts.

Agilent Feature Extraction software (version 11.0.1.1) was used to analyze acquired array images. Quantile normalization and subsequent data processing were performed with GeneSpring GX v12.1 software package (Agilent Technologies). Differentially expressed lncRNAs and mRNAs were identified through fold change/*p* value/FDR filtering (fold change ≥ 2.0, a *P* value ≤ 0.05, and FDR ≤ 0.05). Hierarchical clustering was performed based on differentially expressed mRNAs and lncRNAs using Cluster_Treeview software. The microarray analysis was performed by KangChen Bio-tech, Shanghai, China.

### Gene ontological and pathway analysis

The Gene Ontology (GO) project provided a controlled vocabulary to describe gene and gene product attributed in any organism (http://www.geneontology.org). The ontology covers three domains: biological process, cellular component, and molecular function. Fisher’s exact test was used to determine whether the overlap between the differentially expressed list and the GO annotation list was greater than that expected by chance. The lower the *P* value was the more significant in the GO term enrichment among differentially expressed genes (*P* value ≤ 0.05 was recommended).

Pathway analysis was a functional analysis that mapped genes to KEGG (Kyoto Encyclopedia of Genes and Genomes) pathways (http://www.genome.jp/kegg/). The *P* value (EASE-score, Fisher *P* value, or hypergeometric *P* value) denoted the significance of the pathway correlated to the condition. The GO and KEGG pathways were analyzed by KangChen Bio-tech, Shanghai, China.

### CNC analyses and ceRNA analyses

A coding/non-coding gene co-expression network using 21 mRNAs and the differentially expressed lncRNAs were constructed. The CNC analysis was based on calculating the Pearson correlation coefficient between the expression of coding and noncoding genes. Two correlated genes were screened based on the Pearson correlation using the selection parameters PCC ≥ 0.99 and FDR < 0.05. The co-expression network was illustrated using Cytoscape (v3.4.0). Analyses were performed by KangChen Bio-tech, Shanghai, China.

The potential miRNA response elements were searched on the sequences of lncRNAs and mRNAs. The miRNA binding sites were predicted by miRcode (http://www.mircode.org/), and the miRNA-mRNA interaction was predicted by Targetscan (http://www.targetscan.org/).

### RNA isolation and qRT-PCR analyses

RNA isolation and qRT-PCR analyses were performed. The primers to amplify SNHG7: forward, 5′-TTGCTGGCGTCTCGGTTAAT-3′; reverse, 5′-GGAAGTC CATCACAGGCGAA-3′; GALNT7: forward, 5-GGTACCAT GGCCTCATGTTG-3; reverse, 5-GCCACCACACTGCCATATCT-3′; miR-34a: forward, 5′-CACGGACTC GGGGCATTTGGAGATTTT-3′; reverse, 5′-CTGTCTAGATCGCTTATCTTCCC CTTGG3′. U6: forward 5′-CTCGCTTCGGCAGCACA-3′; reverse 5′-AACGCTT CACGAATTTGCGT-3′; GAPDH: forward 5′-AACGTGTCAGTGGTGGACCTG-3′; reverse, 5′-AGTGGGTGTCGCTGTTGAAGT-3′; miR-34a was normalized to U6, lncRNA-SNHG7 and mRNA expression data were normalized to GAPDH. The relative expression was calculated using the 2-ΔΔCT method.

### Plasmids, oligonucleotides, siRNA, transfection, and dual luciferase assay

SNHG7 pcDNA3.1 vector (SNHG7), GALNT7 pcDNA3.1 vector (GALNT7), and empty vector (vector) were subcloned into the expression vector pcDNA3.1 (Invitrogen, USA). MiR-34a mimic, negative control oligonucleotides (miR-NC), miR-34a inhibitor, negative control oligonucleotide (NC inhibitor), small interfering RNA of SNHG7 or GALNT7 (siSNHG7, siGALNT7), and scramble siRNA of SNHG7 or GALNT7 (siSCR) were purchased from RiboBio (Guangzhou, China). The cells were seeded into six-well plates, and transfection was performed using Lipofectamine 2000 (Invitrogen, Carlsbad, CA, USA) according to the manufacturer’s instruction. ShSCR and shSNHG7 were purchased from RiboBio (Guangzhou, China) and constructed into CRC cell lines for further in vivo experiments. The transfection efficiency was evaluated by fluorescence microscopy by calculating the percentage of fluorescein-labeled cells.

Cells were cultured overnight until 70–80% confluence. Next, cells were co-transfected with pcDNA3.1 SNHG7-wt, pcDNA3.1 SNHG7-mut, pcDNA3.1 GALNT7-wt or pcDNA3.1 GALNT7-mut was transfected into HEK-293T cells together with miR-34a mimic or the control, respectively. Lipofectamine 2000 (Invitrogen Co., Carlsbad, CA, USA) was used according to the manufacturer’s instructions. After 48 h, cells were harvested for luciferase detection using the dual-luciferase reporter gene assay system (Promega, Madison, WI, USA). All values were obtained from at least three independent repetitions of the transfection.

### RNA immunoprecipitation (RIP) assay

RIP assay was performed using the Magna RIP™ RNA Binding Protein Immunoprecipitation Kit (Millipore, Bedford, MA, USA). Cells were collected and lysed in complete RIPA buffer containing a protease inhibitor cocktail and RNase inhibitor. Next, the cell extracts were incubated with RIP buffer containing magnetic bead conjugated with human anti-Ago2 antibody (Millipore) or mouse immunoglobulin G (IgG) control. The protein was digested with proteinase K, and subsequently, the immunoprecipitated RNA was obtained. The purified RNA was finally subjected to a qRT-PCR analysis to demonstrate the presence of the binding targets.

### Ethynyldeoxyuridine (Edu) analysis

Edu detection kit (KeyGENBioTECH, Nanjing, China) was used to assess cell proliferation. Cells were cultured in 96-well plates at 4 × 10^4^ cells/well. Twenty-micromolar Edu labeling media was added to the 96-well plates, and they were then incubated for 2 h at 37 °C under 5% CO_2_. After treatment with 4% paraformaldehyde and 0.5% Triton X-100, the cells were stained with the anti-Edu working solution.

### Viability assay, colony formation assay, and tumorigenicity assays in nude mice

The cell viability was monitored using the Cell Counting Kit-8 (CCK8) according to the manufacturer’s protocol. Measured the OD (optical density) value by microplate computer software (Bio-Rad Laboratories, Hercules, CA). Absorbance at 450 nM (A450) was read on a microplate reader (168–1000 Model 680, Bio-Rad).

Colony formation assay was performed to measure the capacity of cell proliferation. Briefly, 1 × 10^3^ cells were plated in six-well plates. After incubated for 12 days, cells were fixed, stained, photographed, and analyzed.

Tumorigenicity assays in nude mice were performed. Briefly, the mice in groups were inoculated subcutaneously with 1 × 10^7^ cells in the right flank with SNHG7, shSNHG7, or control. Tumor volumes were calculated by using the equation volume (mm^3^) = *A* × *B*^2^/2, where *A* is the largest diameter, and *B* is the perpendicular diameter.

### Cell cycle analysis and apoptosis analysis

Cell cycle and apoptosis analysis were cultured as previously described [16].

### Wound healing, transwell migration, transwell invasion, and endothelial tube formation assay

Wound healing assay was performed to measure the capacity of cell migration. Detailed description of wound healing assay was cultured as previously describe [16]. The results were photographed using an inverted microscope (Olympus, Japan) and analyzed by software IPP Image-Pro Plus 6.0.

Transwell assay was performed using Boyden chambers containing a transwell membrane filter (Corning, New York, USA). Transwell assay analysis was cultured as previously described [16]. Evaluation of invasive capacity was performed by counting invading cells under a microscope (40 × 10). Five random fields of view were analyzed for each chamber.

Detailed description of angiogenesis assay was cultured as previously described [16] and analyzed by ImageJ software (National Institutes of Health, Bethesda, MD).

### Western blot analysis

Western blot analysis was cultured as previously described [16]. GALNT7 monoclonal antibody (1:1000, Abcam, Cambridge, UK), PI3K, p-PI3K Tyr458, Akt, p-Akt Ser473, mTOR, p-mTOR Ser2248 antibody (1:1000 Proteintech, Chicago, USA). Immunoreactive bands were visualized using ECL Western blotting kit (Amersham Biosciences, Buckinghamshire, UK) and were normalized to GAPDH.

### Statistical procedures

The data were presented as the mean ± SD. Comparison between two groups was assessed using an unpaired two-tailed t test. A one-way analysis of variance, the chi-square test, and the Fisher’s exact test were performed. The survival curves were calculated using the Kaplan-Meier method, and the differences were assessed by a log-rank test. The Cox proportional hazards model was used to determine the independent factors. *p* value < 0.05 was considered to be statistically significant. All results were reproduced across triplicate experiments. Statistical analyses were carried out using GraphPad Prism (GraphPad Software, Inc., USA).

## Results

### Expressional profiles of lncRNAs and mRNAs in CRC cell lines

**Fig. 1:**
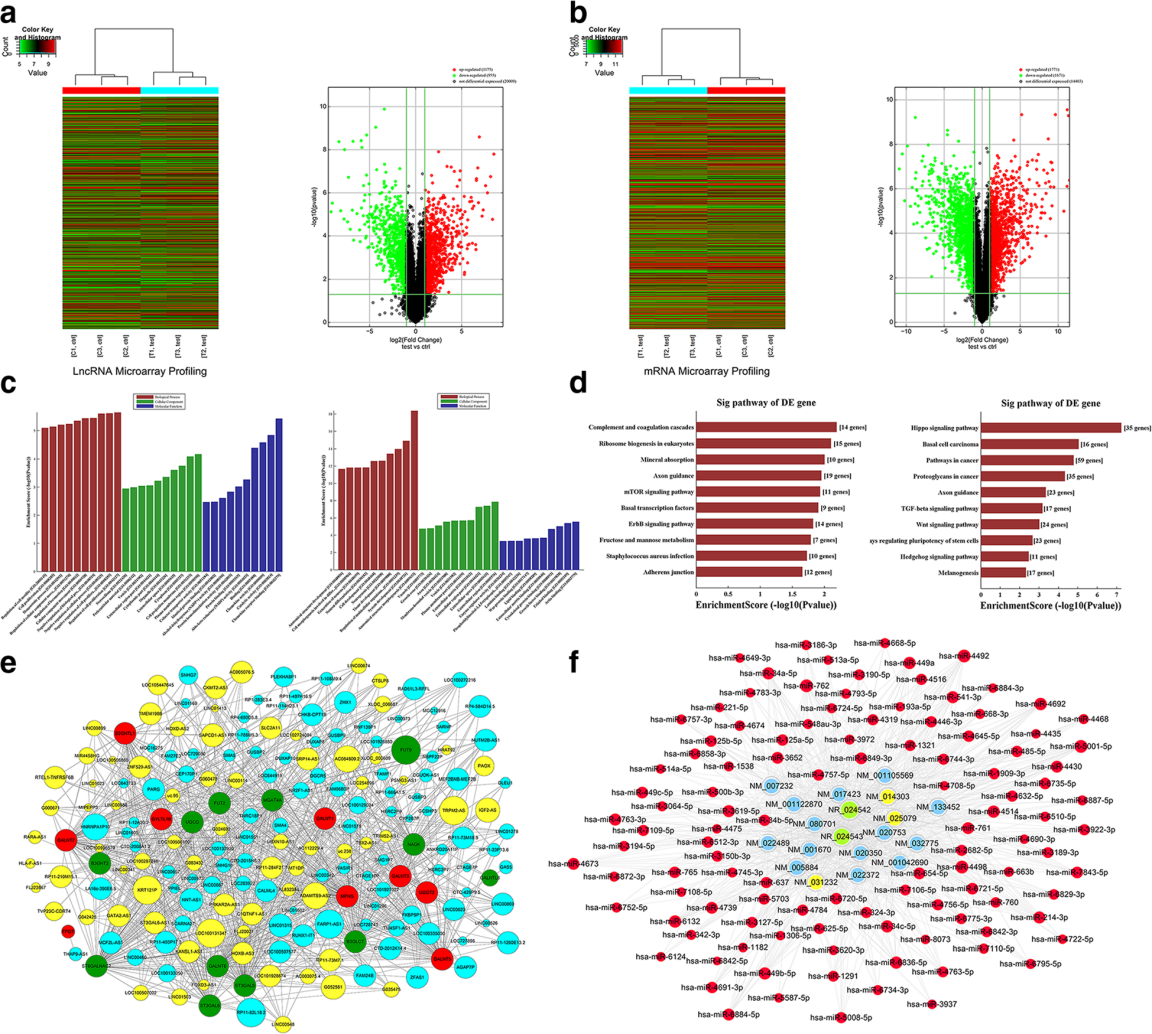
The expressional profiles of lncRNA and mRNA in CRC cell lines. **a**, **b** Differential expression of lncRNAs and mRNAs were detected. **a** and **b** right panels showed the volcano plots for SW620 vs SW480. **c** Go annotation of mRNAs with the top 10 enrichment scores covered domains of biological processes, cellular components and molecular functions. **d** KEGG pathway enrichment analysis of mRNAs with the top 10 enrichment scores was shown. **e** CNC network was shown. Blue and yellow parts represented upregulated and downregulated lncRNAs, respectively. Red and green parts meant upregulated and downregulated mRNAs. **f** ceRNA network was shown. Red color meant microRNAs. Light-blue color meant mRNAs. Light-green color meant lncRNA-SNHG7. Light-yellow color meant predicted circular RNAs

Microarray analysis revealed lncRNAs and mRNAs expression in CRC cells. After screening (fold change ≥ 2.0, *p value < 0.05), 2130 lncRNAs (1175 were upregulated, and 955 were downregulated) notably changed in metastatic cell SW620 relative to primary cell SW480 (Fig. 1a). A total of 3442 mRNAs (1771 were upregulated, and 1671 were downregulated) significantly changed (Fig. 1b). The top 10 upregulated and downregulated lncRNA/mRNAs were listed in Table 1, respectively. Function of upregulated lncRNAs would be discussed.

**Table 1:** The top 10 upregulated and downregulated lncRNAs

Upregulated lncRNAs			Downregulated lncRNAs		
Sequence name	Gene symbol	Fold change	Sequence name	Gene symbol	Fold change
NR_024543	SNHG7	19.3	NR_033203	HOXB-AS3	32.1
NR_038108	SNHG16	18.5	T105469	G024903	25.4
T117110	G027615	17.2	NR_033877	LINC00548	19.8
T117111	G027616	15.8	T377350	G089269	14.8
ENST00000606186	RP4-680D5.8	9.1	NR_039989	MIR4458HG	11.0
ENST00000430859	AC005083.1	9.0	NR_110078	UBXN10-AS1	10.7
NR_109758	LOC644919	8.3	NR_110845	LOC101928674	10.7
TCONS_00019861	XLOC_009079	7.2	NR_120685	LINC01503	10.5
ENST00000602939	RP11-395P17.11	7.1	T232938	G053602	10.1
NR_034119	LINC00460	6.9	NR_034121	CKMT2-AS1	9.2
Upregulated mRNAs			Downregulated mRNAs		
Gene symbol	Fold change		Gene symbol		Fold change
LCP1	90.2		IGFBP3		59.3
S100P	60.6		GLI3		56.5
CNN3	50.8		PTGR1		46.2
TPM2	46.6		ISM2		44.3
GALNT7	43.7		ZAP70		37.7
GALNT1	40.6		RGCC		33.1
CA9	39.9		ARL4C		33.1
GYLTL1B	34.4		CADM1		32.9
GPX2	32.0		TBC1D4		30.8
MFNG	31.9		KRT85		27.8

GO analysis showed that the aberrant mRNA mainly took part in following biological processes in CRC. The upregulated transcripts involved in regulation of cell proliferation, plasma membrane region, and chemokine receptor binding (Fig. 1c). KEGG pathway enrichment analysis for significantly dysregulated mRNAs was useful to reveal related pathways and molecular interaction. These results demonstrated that the upregulated and downregulated mRNAs were associated with 10 pathways (Fig. 1d). MTOR pathway was one of the most upregulated pathways.

Gene co-expression network was constructed to study the relationship between the lncRNAs and mRNAs. One hundred ninety-one lncRNAs (116 were upregulated and 76 were downregulated) and 21 mRNAs (9 were upregulated and 12 were downregulated) were involved in the co-expression network (Fig. 1e). The CNC networks indicated that mRNA was correlated with lncRNAs. CeRNA hypothesis showed RNA transcripts could crosstalk by competing for miRNAs. The ceRNA network of lncRNA-SNHG7 was first built by integrating expression profiles and regulatory relationships of mRNAs, miRNAs, and SNHG7 (Fig. 1f).

### LncRNA SNHG7 is upregulated in CRC tissues and cell lines and correlated with poor progression

**Fig. 2:**
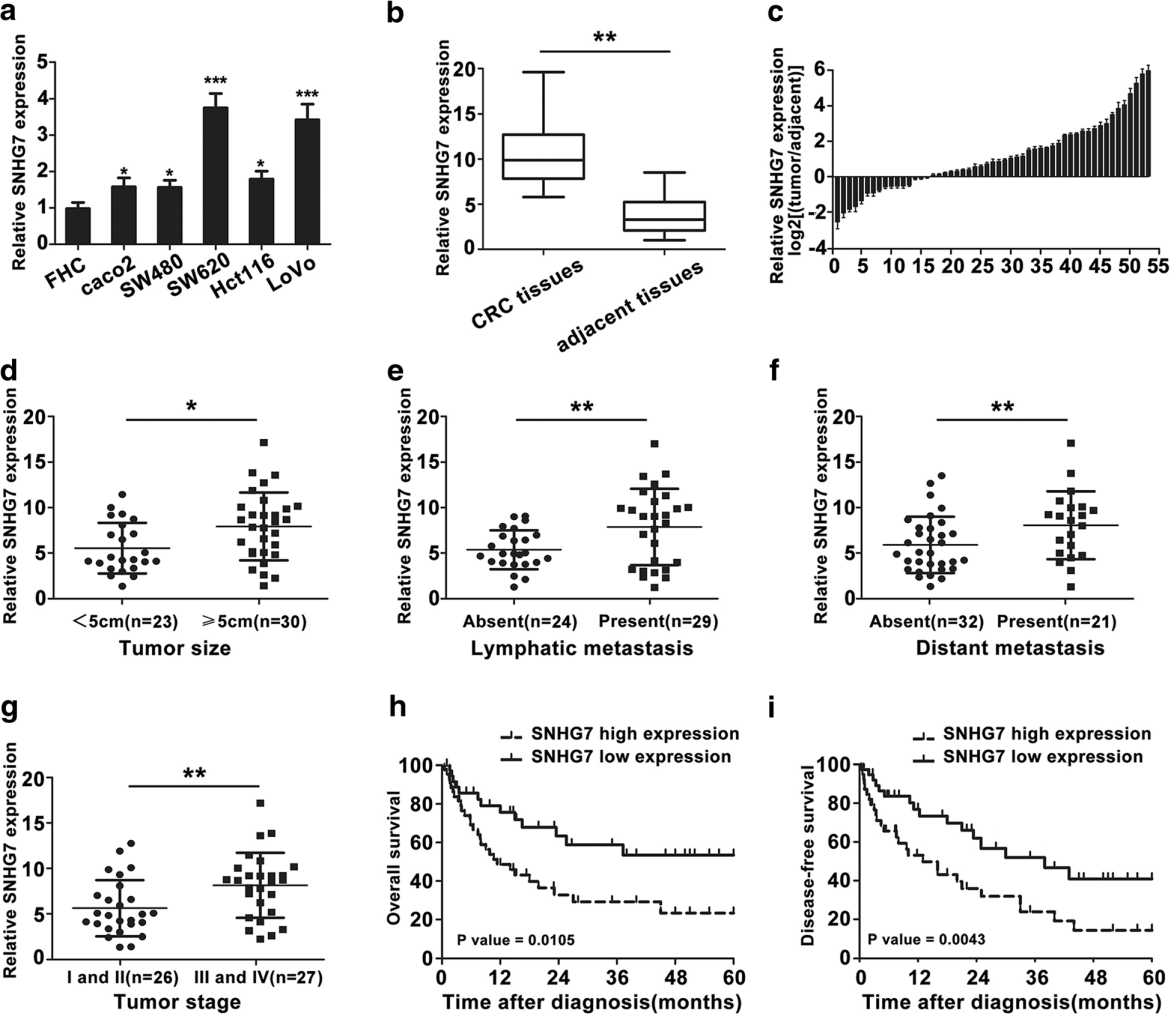
Expression of SNHG7 in CRC tissues, CRC cell lines, and its clinical significance. **a** Expression of SNHG7 in CRC cells was analyzed by qRT-PCR. **b** SNHG7 expression in CRC tissues were determined (cohort 2, *n* = 70). **c** Relative expression of SNHG7 in CRC tissues in comparison with non-tumor tissues was analyzed (cohort 1, *n* = 53). **d**–**g** The correlation between SNHG7 expression and clinical pathological features was shown. SNHG7 upregulation was correlated with larger tumor size, lymphatic metastasis, distant metastasis, and advanced pathological stage in CRC. **h**, **i** Kaplan–Meier OS and DFS rates of 53 patients were shown. The error bars in graphs represented SD. Each experiment was repeated thrice. *P < 0.05, **P < 0.01, ***P < 0.001

The microarray results showed that SNHG7 level was significantly higher in SW620 than in SW480 cells. To further explore SNHG7 expression, CRC cell lines were validated. SNHG7 expression was significantly higher in SW620 cells than in SW480 cells (Fig. 2a). In parallel, SNHG7 level was also higher in CRC tissues than that in adjacent tissues (Fig. 2b, c).

In order to examine the correlation between SNHG7 level and clinical pathological features, we stratified 53 tumor tissue samples (cohort 1) into high and low expression groups (Table 2) and found that SNHG7 expression was significantly correlated with tumor size (< 5 vs. ≥ 5 cm, *P* = 0.025), lymphatic metastasis (absent vs. present, *P* = 0.002), distant metastasis (absent vs. present, *P* = 0.004), and tumor stage (I/II vs. III/IV *P* = 0.002, Fig. 2d–g). Multivariate analysis indicated that SNHG7 expression (95% CI 1.035–5.268; *P* = 0.030), lymphatic metastasis (95% CI 0.869–4.012; *P* = 0.045), distant metastasis (95% CI 1.586–5.275; *P* = 0.011), and tumor stage (95% CI 1.301–6.684; *P* = 0.002) were independent predictors of the prognosis of CRC (Table 3).

**Table 2:** Relationship between SNHG7 expression and clinicopathologic parameters of 53 colorectal cancer patients (cohort 1)

Characteristics	Number of case	SNHG7 expression		*P* value
		High (*n* = 30) %	Low (*n* = 23) %	
	53			
Age (years)				0.477
< 60	27	14	13	
> 60	26	16	10	
Gender				0.337
Male	33	17	16	
Female	20	13	7	
Tumor size				0.025*
< 5 cm	23	9	14	
≥ 5 cm	30	21	9	
Tumor location				0.745
Colon	29	17	12	
Rectum	24	13	11	
Depth of invasion				0.194
T1,T2	34	17	17	
T3,T4	19	13	6	
Histologic grade				0.206
Well/moderately	27	13	14	
Poorly/others	26	17	9	
Lymphatic metastasis				0.002**
Absent	24	8	16	
Present	29	22	7	
Venous invasion				0.132
Absent	27	18	9	
Present	26	12	14	
Nervous invasion				0.414
Absent	31	19	12	
Present	22	11	11	
Distant metastasis				0.004**
Absent	32	13	19	
Present	21	17	4	
Tumor stage*				0.002**
I and II	26	9	17	
III and IV	27	21	6	

*Tumor stage was obtained according to the TNM criteria

**P<0.05

**Table 3:** Univariate and multivariate Cox regression analyses of clinicopathological features association with prognosis of 53 CRC patients (cohort 1)

Variables	Subset	Univariate analysis		Multivariate analysis	
		HR (95% CI)	*P* value	HR (95% CI)	*P* value
Age (years)	< 60 vs. ≥ 60	1.379(0.525–2.484)	0.495	–	–
Gender	Male vs. female	1.514(0.701–2.916)	0.410	–	–
Tumor size	< 5 cm vs. ≥ 5 cm	2.438(1.003–5.105)	0.062	–	–
Tumor location	Colon vs. rectum	1.028(0.712–1.582)	0.875	–	–
Histologic grade	Well/moderately vs. poorly/others	1.847(0.571–2.896)	0.289	–	–
Depth of invasion	T1,T2 vs. T3,T4	2.561(0.911–4.616)	0.124	–	–
Lymphatic metastasis	Absent vs. Present	3.054(1.008–5.531)	0.028*	2.313(0.869–4.012)	0.045*
Venous invasion	Absent vs. Present	1.478(0.491–3.292)	0.353	–	–
Distant metastasis	Absent vs. Present	3.681(1.308–5.928)	0.004*	3.163(1.586–5.275)	0.011*
Tumor stage	I-II vs. III-IV	4.422(1.849–8.241)	< 0.001*	3.549(1.301–6.684)	0.002*
SNHG7 expression	Low vs. high	3.317(1.331–5.609)	0.008*	2.924(1.035–5.268)	0.030*

*HR* hazard ratio, *CI* confidence interval

*P<0.05

To understand the prognostic value of SNHG7, the overall survival (OS) and disease-free survival (DFS) rates were analyzed. High expression of SNHG7 had poorer prognosis than low expression of SNHG7 (*P* = 0.0105, OS, and *P* = 0.0043, DFS, Fig. 2h, i) by Kaplan-Meier and log-rank test analyses. These results implied that SNHG7 overexpression might be useful for the novel prognostic markers in CRC.

### SNHG7 mediates CRC cell proliferation, cell cycle progression, and apoptosis in vitro

**Fig. 3:**
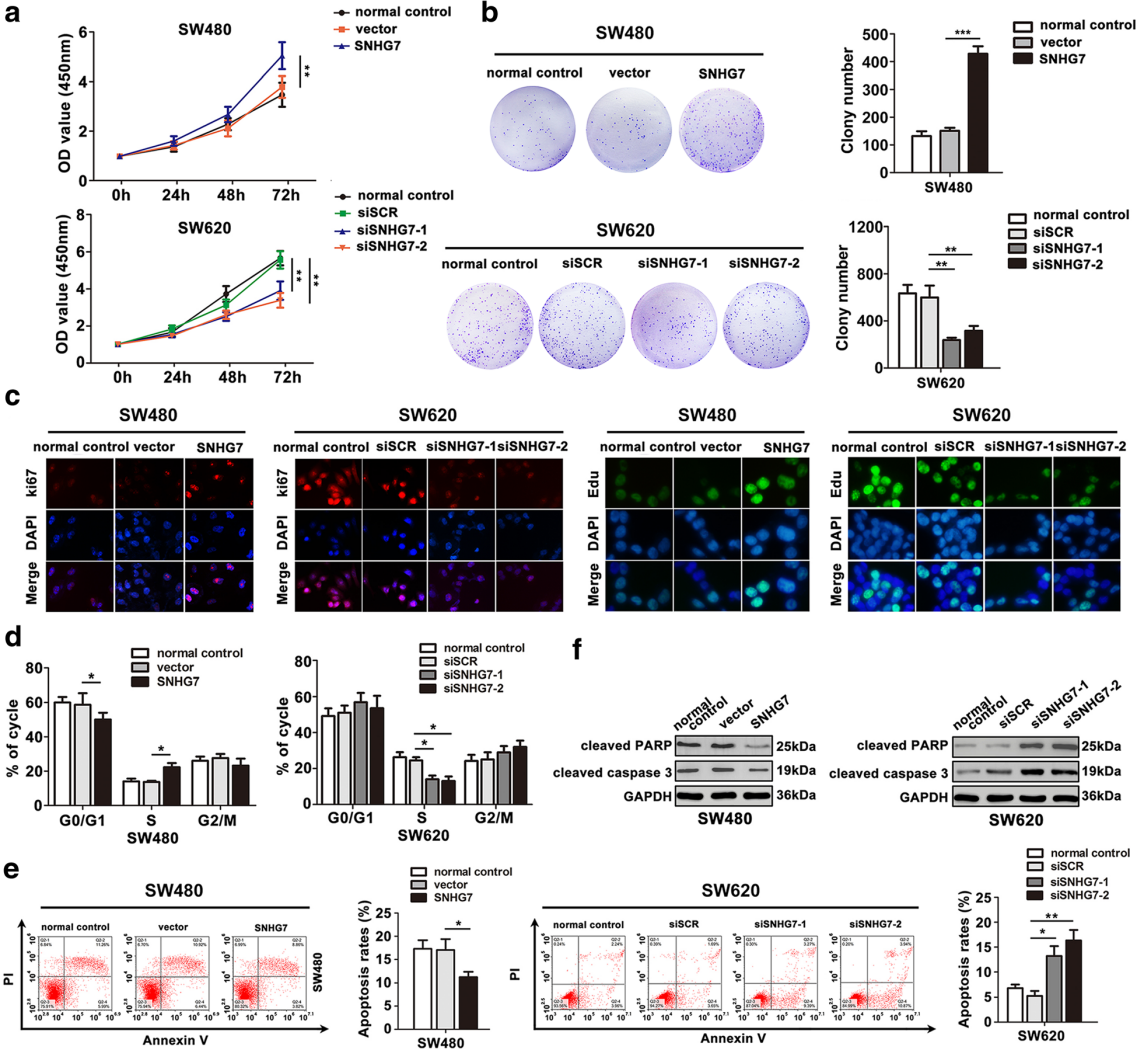
SNHG7 mediates cell proliferation, cell cycle, and apoptosis of CRC cells in vitro. **a**–**c** Cell proliferation was analyzed by CCK-8 assay, colony formation assay, and immunofluorescence analysis with ki67 and Edu. **d** The influence of CRC cell lines in S phase transfected with SNHG7 or siSNHG7 was investigated by FACS analysis. **e** Apoptosis rates of CRC cells transfected with SNHG7 or siSNHG7 was detected by FACS analysis. **f** Relative cleaved PARP and cleaved caspase-3 expression following transfected or silencing SNHG7 in CRC cells were analyzed by Western blot. The error bars in graphs represented SD, and each experiment was repeated thrice. *P < 0.05, **P < 0.01, ***P < 0.001

To investigate the biological significance of SNHG7 in CRC progression, the cell proliferation was analyzed. Following SNHG7 overexpression, the proliferative capability of CRC cell lines was increased using CCK8 assay (Fig. 3a), clone formation (Fig. 3b), and Edu staining (Fig. 3c). Knockdown of SNHG7 attenuated the proliferative ability. In addition, the cell cycle was analyzed following SNHG7 overexpression or silencing by fluorescence-activated cell sorting (FACS). G1/S phase was driven in SW480 cell transfected with SNHG7, whereas stalled G1/S was observed in SW620 cell transfected with siSNHG7 in Fig. 3d. The apoptosis cells were decreased in transfected with SNHG7 group, while in transfected with siSNHG7, SW620 cells had a significantly higher percentage of Annexin V-positive cells by FACS analysis (Fig. 3e). Furthermore, upregulation of SNHG7 led to reduced levels of cleaved caspase 3 and cleaved PARP in SW480 cells, and opposite trend was found in SW620 cells transfected with siSNHG7 compared with siSCR (Fig. 3f). These data indicated that SNGH7 promoted cell proliferation, facilitated cell cycle progression, and inhibited apoptosis in vitro.

### SNHG7 promotes CRC cell migration, invasion, vasculogenic mimicry in vitro, and proliferation in vivo

**Fig. 4:**
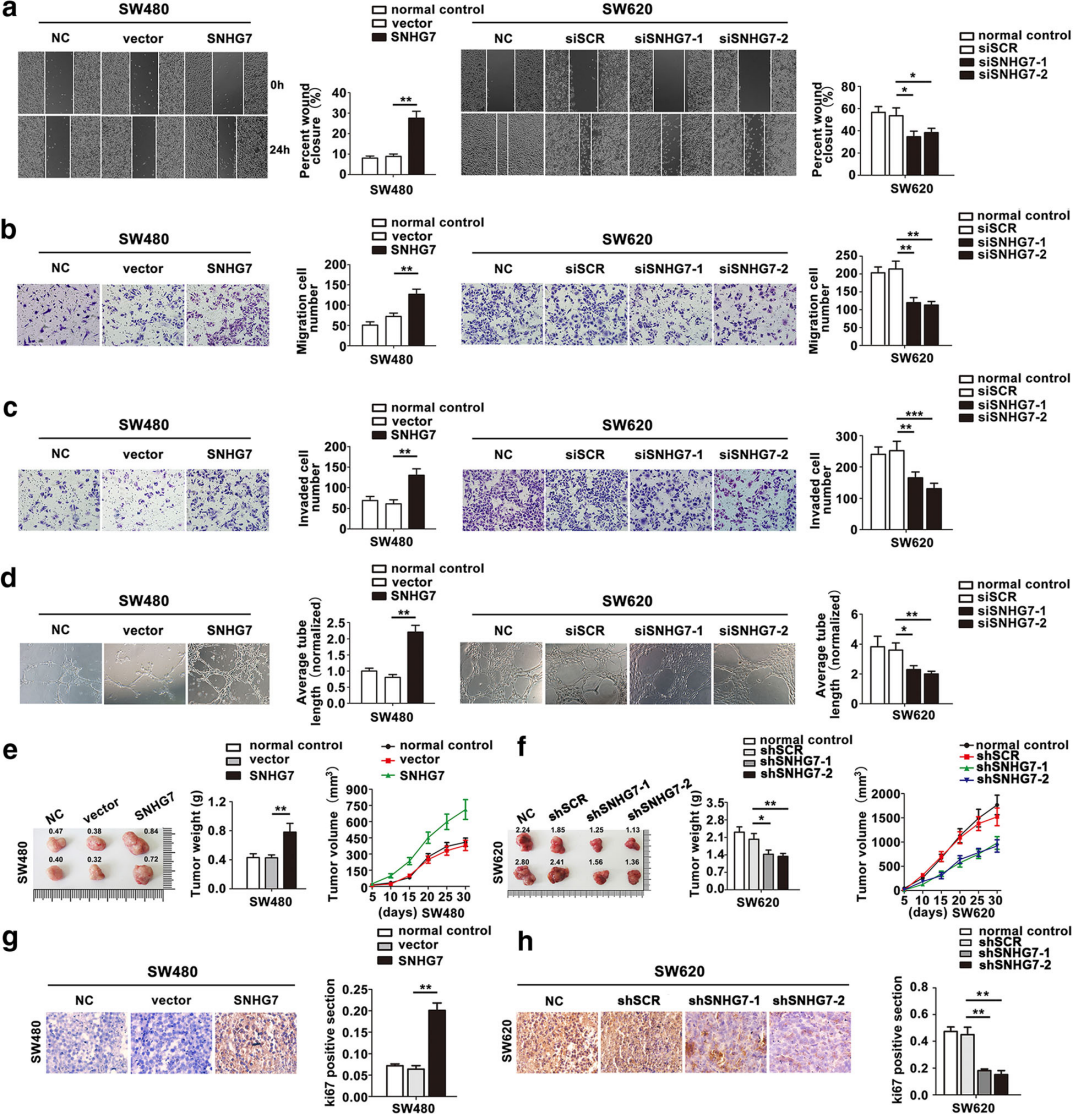
SNHG7 promotes cell migration, invasion, vasculogenic mimicry of CRC cells in vitro, and proliferation in vivo. **a**, **b** The cell migration rates were determined by performing wound healing and transwell assay. **c** Transwell invasion assays were performed. **d** The abilities of neovascularization were determined by endothelial tube formation assay. **e** Effects of SNHG7 overexpression on tumor growth in vivo. Left: images of tumors in nude mice injected with SNHG7-overexpressed SW480 cells. Middle: tumor weights. Right: tumor growth curves. **f** Effects of SNHG7 knockdown on tumor growth in vivo. Left: images of tumors in nude mice injected with shSNHG7 SW620 cells. Middle: tumor weights. Right: tumor growth curves. **g**, **h** Ki67 was detected by IHC assay. The error bars in all graphs represented SD, and each experiment was repeated thrice. *P < 0.05, **P < 0.01, ***P < 0.001

To measure the migratory and invasive ability of CRC cells, wound healing and transwell assays were utilized. The results showed that the ability of migration and invasion were increased in SW480 cell transfected with SNHG7, while the migratory and invasive ability were decreased in SW620 cell transfected with siSNHG7 (Fig. 4a–c).

It is well known that new blood vessel is essential in tumor development. The endothelial tube formation assay was used to assess the ability of SNHG7 in CRC cell progression. The results showed that neovascularization rate was increased after transfecting with SNHG7 or decreased after transfecting with siSNHG7 compared with their negative controls (Fig. 4d).

To confirm whether SNHG7 affect CRC tumorigenesis, SW480 cells (transfected with SNHG7 or NC) and SW620 cells (transfected with shSNHG7 or shSCR) were inoculated into nude mice. As shown in Fig. 4e, f, tumor growth in SNHG7 group was significantly faster than that in NC group, while tumor growth was slower in shSNHG7 group than that in shSCR group. Furthermore, the average tumor weight was obviously changed than their corresponding control group. In order to further validate the growth ability mediated by SNHG7 in vivo, the tumor tissues were for IHC staining with ki67 antibody. These results were consistent with our above data that SNHG7 overexpression enhanced the growth and downregulation of SNHG7 decreased the tumor growth of CRC cell lines (Fig. 4g, h). Collectively, these data demonstrated that SNHG7 promoted CRC cell progression.

### SNHG7 is a direct target of miR-34a and regulates GALNT7 expression in CRC cell lines

**Fig. 5:**
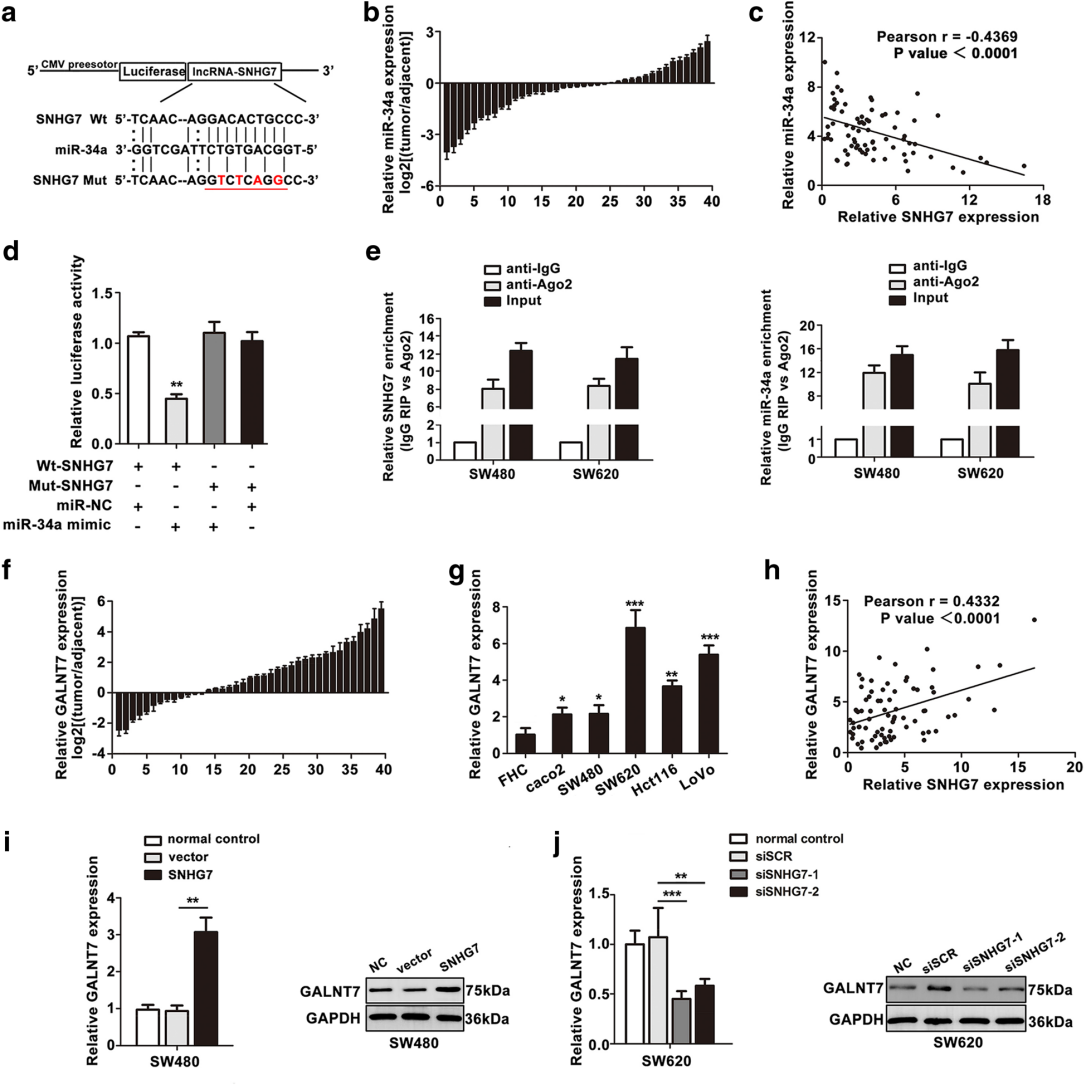
SNHG7 is a direct target of miR-34a and regulates GALNT7 expression in CRC cells. **a** Sequence alignment of miR-34a with the binding sites in the wild-type and mutant-type regions of SNHG7 was shown. **b** miR-34a expression in CRC tissues were determined. **c** The negative relevance between SNHG7 and miR-34a was revealed by Pearson’s correlation curve. **d** The relative luciferase activity of 293T cells was tested after co-transfection with SNHG7 wide-type and miR-34a mimic. **e** RIP assay was performed, and the co-precipitated RNA was subjected to qRT-PCR. RNA levels were presented as fold enrichment in Ago2 relative to IgG immunoprecipitates. **f**, **g** Relative GALNT7 expression of CRC tissues and CRC cells were analyzed. **h** The positive relevance between SNHG7 and GALNT7 expression was revealed by Pearson’s correlation curve. **i** GALNT7 level in SW480 cells transfected with SNHG7 was shown. **j** GALNT7 level in SW620 cells transfected with siSNHG7 was shown. The error bars in graphs represented SD, and each experiment was repeated thrice. *P < 0.05, **P < 0.01, ***P < 0.001

Recently, accumulating evidence has suggested that lncRNA has an inhibitory effect on miRNA expression and activity [17]. CeRNA analysis and bioinformatics software (Starbase v2.0) were used to predict the potential miRNA binding sites in SNHG7. We found that miR-34a was among the numerous possible targets of SNHG7 (Fig. 5a). MiR-34a level was much lower in CRC tissues than that in adjacent tissues (Fig. 5b). Pearson correlation coefficient analysis showed a significant negative correlation between SNHG7 and miR-34a in CRC tissues (Fig. 5c). The luciferase activity of wt-SNHG7 was significantly reduced by miR-34a mimic (Fig. 5d). In contrast, the luciferase activity of mut-SNHG7 experienced no statistical changes. These findings indicated that there were interaction between miR-34a and the binding sites of SNHG7.

The previous study indicated that lncRNAs might act as the sponge of miRNAs and function through binding the miRNAs and Argonaute 2 (Ago2) [18, 19]. According to the bioinformatics software, the sites of SNHG7/miR-34a could also bind the protein Ago2 protein. So, RIP assay was performed in SW480 and SW620 cell extracts utilizing the antibody against Ago2. SNHG7 and miR-34a expression were detected by qRT-PCR. The results illustrated that both SNHG7 and miR-34a were enriched in the Ago2 pellet relative to control IgG immunoprecipitate (Fig. 5e), suggesting that SNHG7 was present in Ago-contained miRNPs, likely through an association with miR-34a.

Gene co-expression networks indicated that SNHG7 was correlated with nine mRNAs (B3GLCT, FUT2, MFNG, MGAT4A, GALNT1, GALNT5, GALNT7, ST3GAL5, ST6GALNAC2). GALNT7 was the highest correlation among these mRNAs in Pearson analysis. GALNT7 level was identified in CRC tissues and metastasis cell lines (Fig. 5f, g), and a positive relationship between the levels of SNHG7 and GALNT7 mRNA was found (Fig. 5h). The expression of GALNT7 mRNA and protein were significantly increased in SW480 cells overexpressing SNHG7 (Fig. 5i). In contrast, GALNT7 level was downregulated in SW620 cells with silencing SNHG7 (Fig. 5j). These results showed that SNHG7 indeed regulated GALNT7 level.

### GALNT7 is a target gene of miR-34a in CRC cell lines

**Fig. 6:**
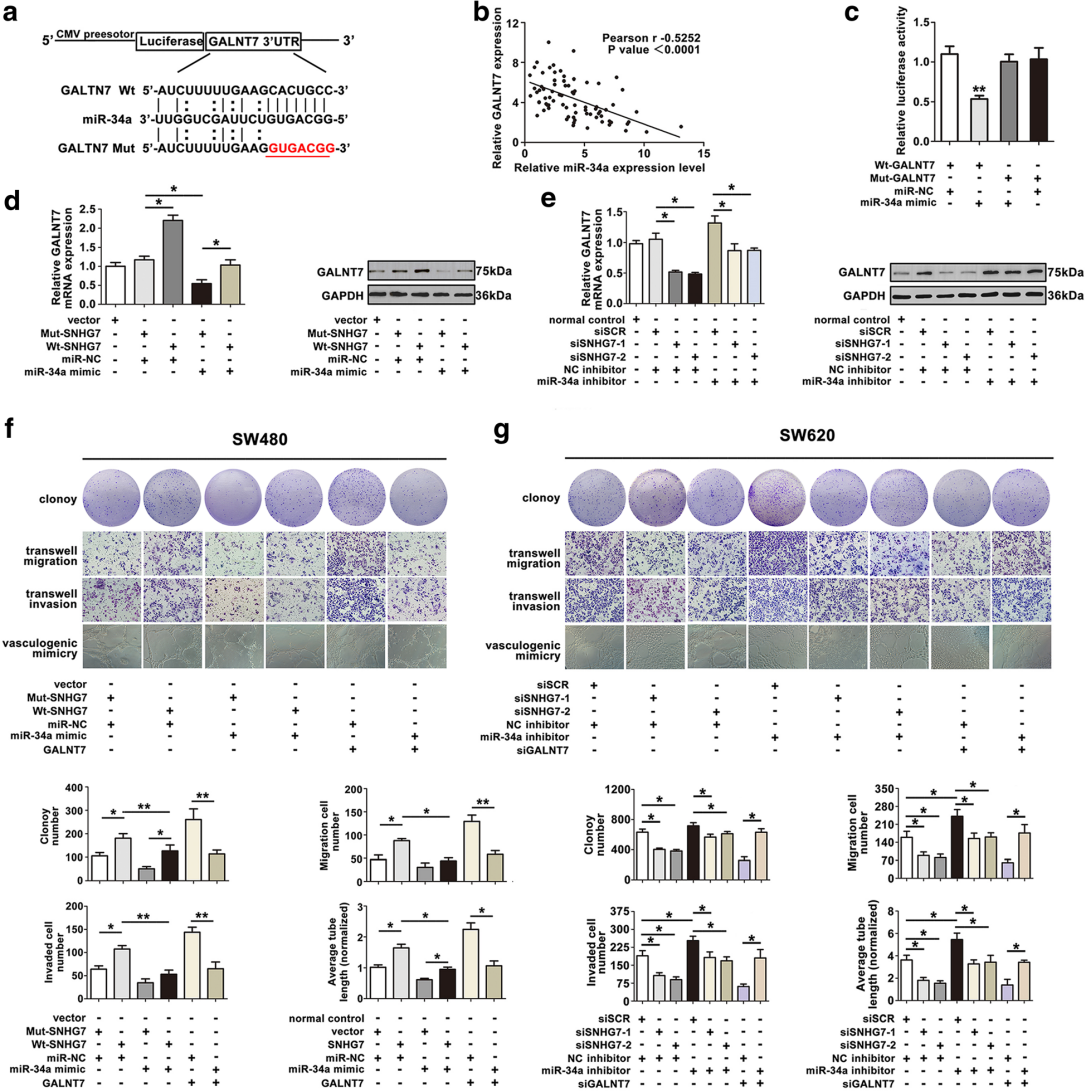
GALNT7 is a target gene of miR-34a in CRC cells. **a** Sequence alignment of miR-34a with the binding sites in the wild-type and mutant-type regions of GALNT7 was shown. **b** The negative relevance between miR-34a and GALNT7 expression was revealed by Pearson’s correlation curve. **c** The relative luciferase activity of 293T cells was tested after co-transfection with GALNT7 wide-type and miR-34a mimic. **d** GALNT7 level in SW480 cells co-tranfected with SNHG7 and miR-34a mimic was analyzed. **e** GALNT7 level in SW620 cells co-tranfected with siSNHG7 and miR-34a inhibitor was shown. **f**, **g** SNHG7 and GALNT7 competing to bind with miR-34a were identified by function screen analysis in SW480 and SW620 cells. The error bars in graphs represented SD, and each experiment was repeated thrice. *P < 0.05, **P < 0.01

Lately, ceRNA hypothesis was proposed to describe the crosstalk of RNA transcripts with each other by using MREs. LncRNAs participate in ceRNA networks and mRNA-miRNA-lncRNA crosstalk [20]. We initially identified GALNT7 as potential target of miR-34a using public prediction algorithms (Target-Scan, miroRNA.org and Starbase v2.0) (Fig. 6a). Then, Pearson analysis showed a negative correlation with miR-34a and GALNT7 in CRC samples (Fig. 6b). Next, the dual luciferase reporter assay revealed that GALNT7 was the direct target of miR-34a (Fig. 6c).

To verify the phenomenon of mRNA and lncRNA competed for the binding of miRNA, GALNT7 expression was analyzed. The GALNT7 level was higher in SW480 cell transfected with Wt-SNHG7 than that transfected with Mut-SNHG7 (Fig. 6d). The influence of Wt-SNHG7 could be reversed by transfecting miR-34a mimic in SW480 cells, which identified that SNHG7 regulated GALNT7 by sponging for miR-34a. SNHG7 knockdown led to a decreased GALNT7 expression, while GALNT7 level could be reversed by co-transfecting siSNHG7 and miR-34a inhibitor in SW620 cells (Fig. 6e).

SNHG7 or GALNT7 and miR-34a mimic were co-transfected into SW480 cells; the ability of proliferation, migration, invasion, and vasculogenic mimicry were recovered (Fig. 6f). By contrary, knockdown of SNHG7 or GALNT7 expression weakened the proliferation and metastasis in SW620 cells. Suppression of miR-34a relieved the reduced level of SNHG7 or GALNT7 (Fig. 6g). All of the outcomes above explained that SNHG7 functioned as a ceRNA to regulate GALNT7 expression by sponging miR-34a in CRC progression.

### SNHG7 activates the PI3K/AKT/mTOR pathway

**Fig. 7:**
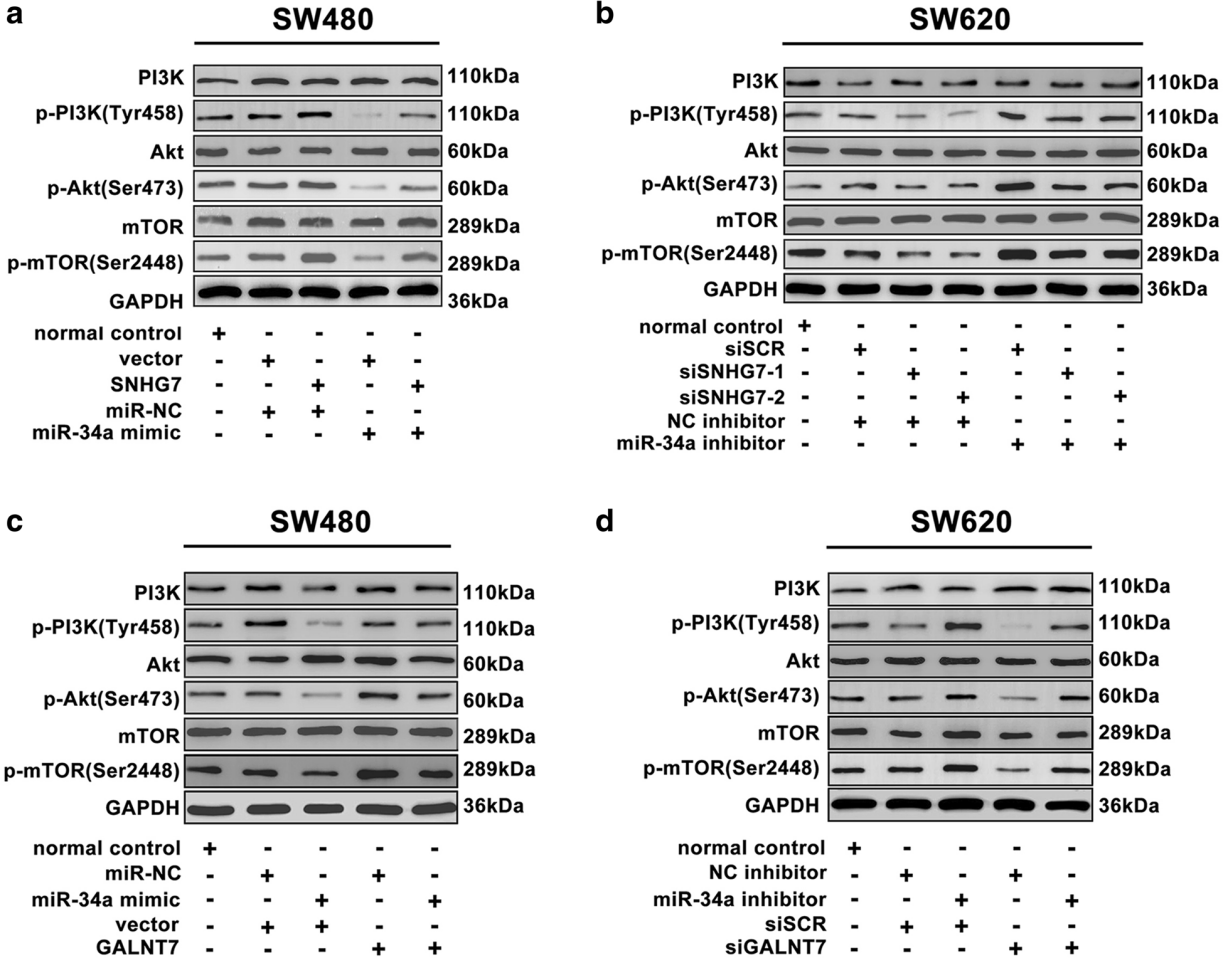
SNHG7 and GALNT7 compete for the binding of miR-34a to activate the PI3K/Akt/mTOR pathway. **a**, **c** The levels of main molecules of PI3K/Akt/mTOR pathway in SW480 cell co-transfected SNHG7, GALNT7 or vector with miR-34a mimic or miR-NC were analyzed. **b**, **d** The levels of main molecules of PI3K/Akt/mTOR pathway in SW620 cells co-transfected siSNHG7, siGALNT7 or siSCR with miR-34a inhibitor or NC inhibitor were shown. The error bars in graphs represented SD, and each experiment was repeated thrice

KEGG pathway enrichment analysis identified that mTOR pathway was one of the most enriched pathways, involved in the development of CRC. In order to figure out the molecular mechanism induced by SNHG7, miR-34a, and GALNT7, the activity of PI3K/AKT/mTOR pathway was detected in the SW480 and SW620 cells. The results showed that high levels of p-PI3K, p-Akt, and p-mTOR were observed in SW480 cells transfected with SNHG7 or GALNT7 than control groups, respectively. The degree of PI3K/Akt/mTOR pathway was decreased in SW480 cell transfected with miR-34a mimic (Fig. 7a, c). Correspondingly, the expression of PI3K/Akt/mTOR pathway proteins was decreased in SW620 cell transfected with siSNHG7 or siGALNT7 (Fig. 7b, d). SW620 cells treated with LY294002 or siSNHG7 exhibited apparently decreased levels of the main signal molecules of PI3K/Akt/mTOR pathway (Additional file 1: Figure S1a). The inhibition of PI3K/Akt/mTOR pathway resulted in decreased proliferation and invasion of SW620 cells (Additional file 1: Figure S1b). These data suggested that the SNHG7, miR-34a, and GALNT7 played an important role in CRC progression via PI3K/AKT/mTOR pathway.

## Discussion

Early diagnosis, especially biomarkers, is an effective means to reduce the mortality of CRC patients. In this study, we explored the possible mechanism of lncRNA-SNHG7, as competing endogenous RNA, modulating GALNT7 by sponging miR-34a in human CRC cell lines.

LncRNAs exerted more complex effects on cell proliferation, differentiation, and epigenetic processes [21, 22]. LncRNA-ATB as a biomarker involved in the progression of CRC [23]. LncRNA-CCAT1-L regulated MYC locus in CRC progression [24]. In this study, we used lncRNA microarray to analyze the composition profiling of lncRNAs in CRC cells. The differences in lncRNA expression, especially SNHG7 were found. SNHG7 was upregulated in CRC tissues and metastatic cell lines. Furthermore, SNHG7 upregulation was correlated with tumor size, lymphatic metastasis, distant metastasis, and tumor stage of clinicopathologic parameters. The OS and DFS of CRC patients with low expression of SNHG7 were longer than that with high expression of SNHG7. Moreover, SNHG7 promoted CRC cell progression both in vitro and in vivo. Recent studies showed that SNHG7 was correlated with breast cancer [9], chromophobe renal cell carcinoma [10], and lung cancer [11]. These data suggested that SNHG7 was CRC progression-related. Further investigation was needed to elucidate the role of SNHG7 in CRC.

CeRNA hypothesis emerged as an alternative function for lncRNAs [25]. The novel regulatory mechanism has been identified in crosstalk between lncRNAs and mRNAs. LncRNA HULC promoted tumorigenesis via the miR-200a-3p/ZEB1 pathway in hepatocellular carcinoma [26]. LncRNA RSU1P2, acting as a ceRNA against let-7a, promoted tumorigenesis in cervical cancer [27]. Our current research observed an inverse correlation between SNHG7 and miR-34a. MiR-34a could bind the SNHG7 by conducting the dual luciferase assay. Also, we found an endogenous interaction between SNHG7 and miR-34a by utilizing RIP assays with the Ago2 antibody in CRC cells. SNHG7 regulated CRC cell progression partially mediated by miR-34a, which was highly correlated with CRC malignancy [28].

Aberrant *O*-glycosylation is a hallmark of metabolic disorders and many cancers. Altered cell-surface *O*-glycoproteins are often implicated in proliferation, invasion, and metastasis [29]. GALNTs were illustrated as the importance in cancer pathogenesis. The GALNT levels vary with cell type, differentiation, and malignant transformation [30, 31]. GALNT12 mutation inactivated the normal function of the GALNT enzyme in initiating mucin type O-linked protein glycosylation in colon cancers [32]. MiRNA cluster controlled glycosylation by targeting GALNTs, responsible for initiating mucin-type O-linked glycosylation [33]. Aberrant glycosylation resulting from GALNT1 involved in melanoma [34], ovarian [35], and bladder cancers [36]. Overexpression of GALNT2 inhibited IGF-l-stimulated growth, migration, and invasion of neuroblastoma cells [37]. GALNT3 was predicted as an independent prognostic factor in renal cell carcinomas [38], and GALNT6 was found to function in pancreatic cancer [39]. Upregulation of GALNT5 played a major role in hepatoblastoma progression [40]. GALNT7, as a downstream target of miR-34a, encoded GalNAc-transferase 7 to participate in laryngeal squamous cell carcinoma [13]. Several studies have reported the function of GALNT7 in the regulation of hepatocellular carcinoma [41] and cervical cancer [14]. These findings suggested that genetic defects in the *O*-glycosylation in part underlied aberrant glycosylation in CRC. In our study, GALNT7 expression was increased in metastatic CRC cells and tumor tissues. Therefore, GALNT7 implicated as a prognostic marker and therapeutic target for CRC.

In recent years, miRNAs could serve functionally as oncogenes or tumor suppressors in cancers. MiR-30e regulated GALNT7 transcripts in cervical cancer [42]. MiR-34a was downregulated in colon cancer specimens compared to normal colonic mucosa [43]. MiR-30a-5p regulated GALNT7 transcripts in renal cell carcinoma [44]. In this study, miR-34a also regulated endogenous GALNT7 expression in CRC cell lines and directly targeted GALNT7. LncRNAs played a part in ceRNA networks and lncRNA-miRNA-mRNA crosstalk. SNHG7 and GALNT7 were constructed in gene co-expression networks, and SNHG7 could regulate GALNT7 level. MiR-34a directly targeted SNHG7-3′UTR. Altered levels of SNHG7, miR-34a, and GALNT7 were associated with progression of CRC cells. Taken together, these data indicated that SNHG7 participated in ceRNA networks and SNHG7-miR-34a-GALNT7 crosstalk played a vital role in CRC progression.

PI3K/Akt/mTOR pathway is known to control the progression of cancer [45]. SNHG7 has potential to influence ribosome biogenesis, which could regulate mTOR transcription [46]. MiR-34a could regulate diffuse malignant peritoneal mesothelioma progression by modulating Akt [47]. CRC cell lines SW480 and SW620 with differently metastatic potential were used in this study. SNHG7, miR-34a, and GALNT7 expression altered between SW480 and SW620 cells. SNHG7 acted as ceRNA to regulate miR-34a availability for the target gene GALNT7, which modulated the PI3K/Akt/mTOR pathway. LY294002 represented the first generation inhibitors with highly potent PI3K-inhibitory property [48]. The inhibition of PI3K/Akt/mTOR pathway by LY294002 or siSNHG7 altered proliferative and invasive abilities of SW620 cells. Therefore, targeting the SNHG7/miR34a/GALNT7 interaction might represent a novel therapeutic application, thus contributing to the metastatic mechanism in CRC patients.

**Fig. 8:**
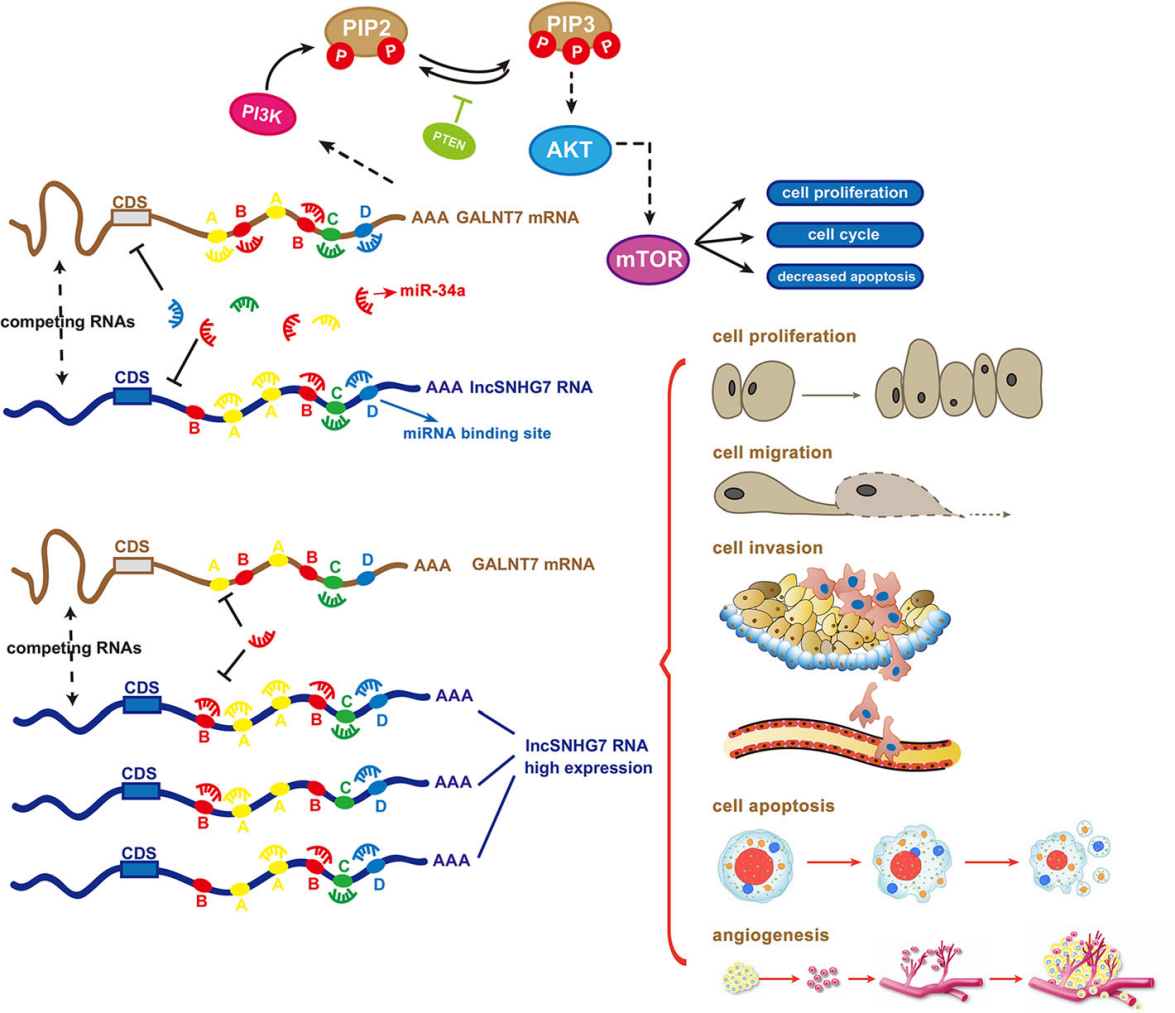
Mechanism for the regulatory function of SNHG7 and miR-34a modulating GALNT7 expression

Our study defined a mechanism for the regulatory function of SNHG7 and miR-34a modulating GALNT7 expression in Fig. 8. The identification of these ceRNAs will undoubtedly enhance our knowledge to clarify the mechanism of SNHG7-miR-34a-GALNT7 axis involving in CRC progression.

## Conclusion

In summary, our study showed that SNHG7 as a ceRNA to regulated GALNT7 by sponging miR-34a in CRC and played the oncogenic role in regulating PI3K/Akt/mTOR pathway. Targeting the SNHG7/miR34a/GALNT7 interaction may represent a novel therapeutic application, thus contributing to better knowledge of the metastatic mechanism in CRC patients.

## Additional file

Additional file 1: **Figure S1.** PI3K/Akt/mTOR pathway inhibition modulates the proliferation and invasion of SW620 cells. (a) SW620 cells were treated LY294002 or siSNHG7. The main molecular expression of PI3K/Akt/mTOR pathway was detected by western blot. (b) LY294002 or siSNHG7 treatment also alleviated proliferation and invsion of SW620 cells. (TIF 2009 kb)

## Abbreviations

3’UTR: 3′-Untranslated region
AGO2: Argonaute 2
ceRNA: Competing endogenous RNA
CNC networks: Coding-non-coding gene co-expression network
CRC: Colorectal cancer
DFS: Disease-free survival
FACS: Fluorescence-activated cell sorting
GALNT7: UDP-*N*-acetyl-α-d-galactosamine: polypeptide-*N*-acetylgalactosaminyltransferase 7
LncRNAs: Long non-coding RNAs
miRNAs: MicroRNAs
MREs: MicroRNA response elements
OS: Overall survival
RIP: RNA immunoprecipitation
SNHG7: Small nucleolar RNA host gene 7
VM: Vasculogenic mimicry
