# Supplementary figures and images for: Long non-coding RNA-SNHG7 acts as a target of miR-34a to increase GALNT7 level and regulate PI3K/Akt/mTOR pathway in colorectal cancer progression
Source: J Hematol Oncol. 2018 Jul 3;11:89. doi: 10.1186/s13045-018-0632-2 (PMC6029165; doi:10.1186/s13045-018-0632-2)

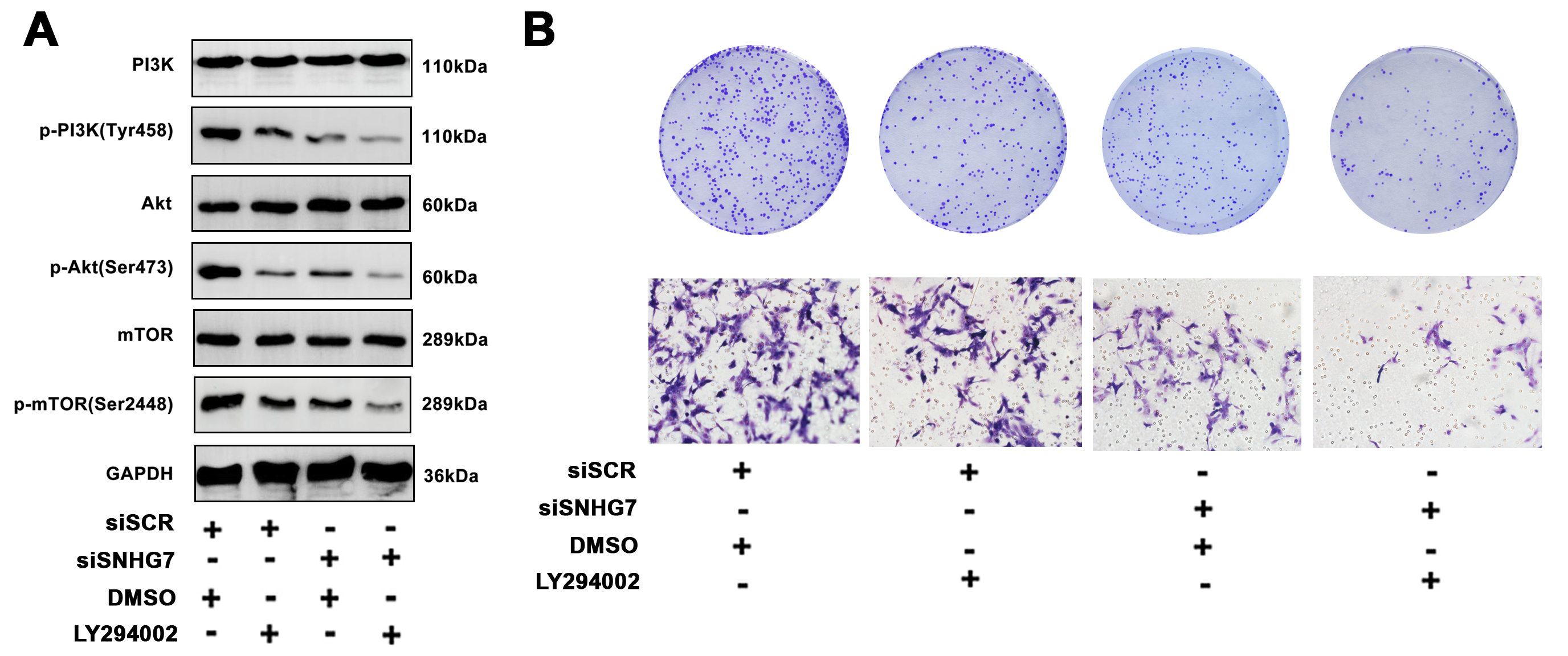

Supplement: Supplementary file 1 — Figure S1. PI3K/Akt/mTOR pathway inhibition modulates the proliferation and invasion of SW620 cells. (a) SW620 cells were treated LY294002 or siSNHG7. The main molecular expression of PI3K/Akt/mTOR pathway was detected by western blot. (b) LY294002 or siSNHG7 treatment also alleviated proliferation and invsion of SW620 cells. (TIF 2009 kb) [file 13045_2018_632_MOESM1_ESM.tif]
